# Pentoxifylline as a Potential Adjuvant Therapy for COVID-19: Impeding the Burden of the Cytokine Storm

**DOI:** 10.3390/jcm10225305

**Published:** 2021-11-15

**Authors:** Wiktoria Feret, Magdalena Nalewajska, Łukasz Wojczyński, Wojciech Witkiewicz, Patrycja Kłos, Violetta Dziedziejko, Andrzej Pawlik

**Affiliations:** 1 Department of Nephrology, Transplantology and Internal Medicine, Pomeranian Medical University, 70-111 Szczecin, Poland; feretwiktoria@gmail.com (W.F.); nalewajska@gmail.com (M.N.); wojczynski@gmail.com (Ł.W.); 2 Department of Cardiology, Pomeranian Medical University, 70-111 Szczecin, Poland; witkiewiczwojciech@gmail.com; 3 Department of Biochemistry and Medical Chemistry, Pomeranian Medical University, 70-111 Szczecin, Poland; patison@pum.edu.pl (P.K.); viola@pum.edu.pl (V.D.); 4 Department of Physiology, Pomeranian Medical University, 70-111 Szczecin, Poland

**Keywords:** COVID-19, SARS-CoV-2, cytokine storm, cytokines, pentoxifylline, pneumonia, ARDS, pulmonary fibrosis

## Abstract

The outburst of inflammatory response and hypercoagulability are among the factors contributing to increased mortality in severe COVID-19 cases. Pentoxifylline (PTX), a xanthine-derived drug registered for the treatment of vascular claudication, has been reported to display broad-spectrum anti-inflammatory and immunomodulatory properties via adenosine A2A receptor (A2AR)-related mechanisms, in parallel to its rheological actions. Prior studies have indicated the efficacy of PTX in the treatment of various pulmonary diseases, including the management of acute respiratory distress syndrome of infectious causes. Therefore, PTX has been proposed to have potential benefits in the treatment of SARS-CoV-2 symptoms, as well as its complications. The aim of this review is to discuss available knowledge regarding the role of PTX as a complementary therapeutic in SARS-CoV-2.

## 1. Introduction

The outbreak of SARS-CoV-2 infection in Wuhan, China, at the turn of 2019 and 2020 has spread across the globe, resulting in a worldwide pandemic. Manifestations of COVID-19 (coronavirus disease 2019) in adults mainly concern the upper-respiratory tract and vary significantly, ranging from asymptomatic to severe viral pneumonia. Critically ill patients present with deterioration in respiratory function, development of acute respiratory distress syndrome (ARDS), and suffer from coagulopathy, all of which contribute to increased mortality. In adolescents, SARS-CoV2 infection can manifest as various non-respiratory symptoms, including late-onset rashes as well as gastrointestinal or ocular disturbances [1]. Emerging evidence proposes a robust inflammatory reaction as a major pathogenic mechanism responsible for severe lung tissue damage in SARS-CoV-2 infection. The cytokine storm phase of SARS-CoV-2 disease most likely results from amplified production of numerous proinflammatory factors, including interleukin 1ß (IL-1ß), interferon γ (INF-γ), monocyte chemoattractant protein 1 (MCP-1), IL-4, IL-7, IL-8, IL-9, IL-10, and tumor necrosis factor α (TNF-α) [2].

Except for tocilizumab, a monoclonal antibody against IL-6 receptors that has been proven to reduce disease progression and lower the rates of hospitalized patients in need of mechanical ventilation [3], no other therapeutic approaches have thus far been introduced for countering the cytokine storm syndrome associated with COVID-19 in common clinical practice. The current public health emergency has created an urge to repurpose already available drugs to treat the SARS-CoV-2-induced inflammatory state. Hence, pentoxifylline (PTX), a drug with an already established safety profile, has been proposed as a potentially beneficial strategy in fighting COVID-19. 

PTX is a xanthine-derived, commercially produced drug approved for the management of intermittent claudication in patients suffering from a chronic occlusive arterial disease of the limbs [4]. In parallel to its rheological actions, PTX has been documented to display anti-inflammatory and immunomodulatory effects, as well as some antithrombotic and antiviral effects. These pluripotent properties could be of great value in the context of the management of SARS-CoV-2 and its complications. 

## 2. The Cytokine Storm

The so-called “cytokine storm” refers to the pathologically up-regulated production of various proinflammatory molecules in response to infection or other external stimuli [5]. This complication involves a loss of negative feedback on immune cells, thus leading to further recruitment of cytokines to the site of inflammation and subsequent organ damage. SARS-CoV-2 pulmonary manifestations are commonly linked to the occurrence of lung tissue dysfunction, such as diffuse alveolar damage, alveolar edema, thickening of alveolar walls, desquamation of pneumocytes, and hyaline membrane formation, all of which are indicative of ARDS [6] and manifest clinically with disturbed ventilation and hypoxemia. Cytokine storm has been proposed as a primary mechanism responsible for the development of ARDS in COVID-19, exceeding the direct cytopathogenic action of the virus itself [7]. Chen et al. have demonstrated increased production of the proinflammatory molecules IL-6, IL-2R, IL-10, and TNF-α in COVID-19, showing that they correlate with disease severity [8]. The IL-6, an important cytokine in viral infections, is further responsible for activation of the coagulation cascade and increased vascular permeability, resulting in an outburst of inflammation [9]. Furthermore, SARS-CoV-2 S protein can induce IL-6 and TNF-α production in murine macrophages in vitro in an NF-κB-dependent manner [10].

**Figure 1:**
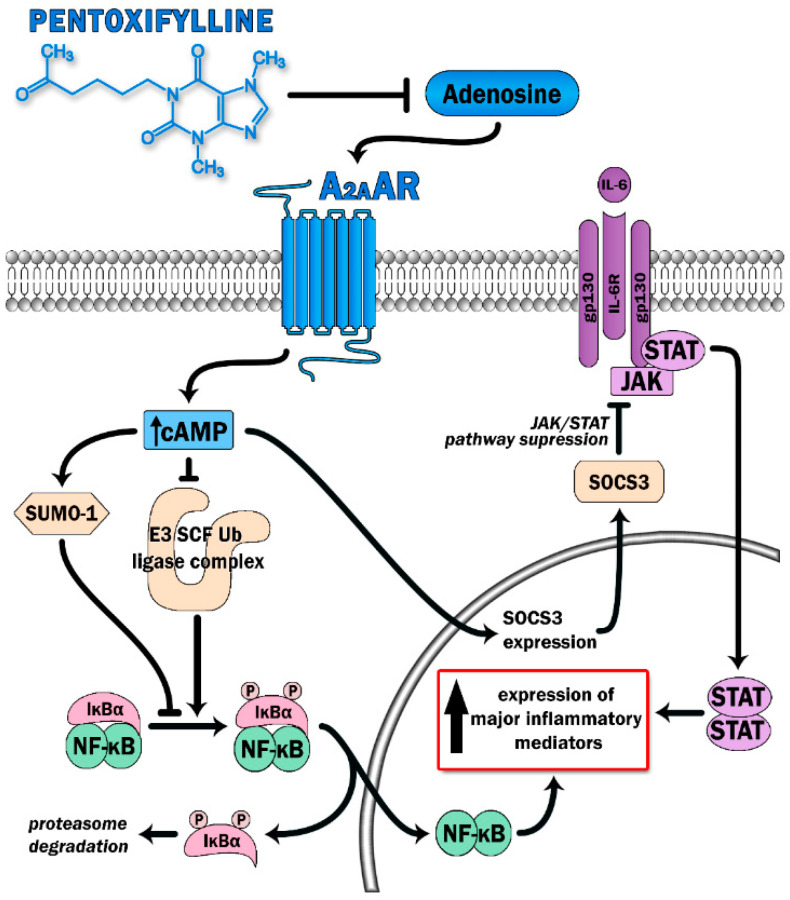
Pentoxifylline is able to dampen activation of two major proinflammatory signaling pathways (the NFκB and JAK-STAT) via antagonist effect on adenosine binding with its receptor (A_2A_AR). Triggering A_2A_AR causes elevation of intracellular cAMP levels. This leads to increase SUMO-1 activity along with inhibition of the E3 SCF Ub ligase complex, which prevents IκBα ubiquitination and degradation, thereby switching off the NFκB pathway. Moreover, high cAMP levels suppress cytokine-mediated JAK/STAT signaling via induction of the inhibitory protein SOCS3.

PTX has been proven to display anti-inflammatory effects and, therefore, was selected as a treatment with potential benefit in countering the COVID-19-mediated cytokine storm. PTX has been considered to boost cAMP levels via the inhibition of phosphodiesterase 4 (PDE-4). The inhibition of the enzyme results in a decreased breakdown of cAMP and further in its increased intracellular levels. Modulation of inflammatory processes by PTX occurs via inhibition of NF-κB, leading to reduced leukocytes–platelets interactions and prothrombotic effects, as well as reduced activation of proinflammatory cytokines and reactive oxygen species [11]. PDE-4 inhibition has already displayed favorable effects in various conditions, i.e., asthma, chronic obstructive pulmonary disease, and idiopathic pulmonary fibrosis (IPF) [12]. However, an additional mechanism of action of PTX has recently been proposed. Adenosine A2A receptors (A2ARs) are 7-pass G-protein-coupled receptors that escalate the activity of adenylate cyclase, resulting in increased production of intracellular cAMP in multiple cells, such as neutrophils, macrophages, T-cells, natural killer cells, endothelial cells, and platelets [13,14]. The ability of A2ARs to diminish inflammatory responses mainly relies upon the deactivation of the two main proinflammatory pathways: the NF-κB and JAK/STAT pathways [13] (Figure 1). Increased levels of cAMP contribute to a reduction in the release of oxidants and cytokines, decreased expression of adhesion molecules, decreased generation of tissue factors, and decreased platelet aggregation [13]. Stimulation of A2ARs further blocks the transition of neutrophils through the lung endothelium, a phenomenon that underlies the pathogenesis of ARDS [13]. PTX potentiates the response of A2ARs to extracellular adenosine, therefore participating in A2AR-related anti-inflammatory pathways Previously, PTX has been proven to reduce lung inflammation and decrease the number and activity of lung neutrophils in rodent models of hemorrhagic shock, when administrated as an adjuvant therapy to standard fluid resuscitation [15]. In the same study, PTX regimens resulted in attenuation of the NF-κB activity, possibly through a cAMP-related mechanism [15]. The capacity of PTX to inhibit TNF-α secretion in vitro has been confirmed through a randomized controlled study of advanced cancer patients diagnosed with ARDS [16]. The study group was characterized by improvements in both mean survival time and the clinical and radiological symptoms of ARDS, without any serious adverse effects [16]. PTX has also been shown to attenuate lung injury and improve mortality rates in mice induced to ARDS by increasing cAMP levels and restoring the Treg/Th17 imbalance, in parallel to decreases in IL-2, IL-6, IL-10, and IL-17 secretion [17]. In a recently published pilot study on patients with a moderate-to-severe SARS-CoV-2 disease course, treatment with PTX led to increases in lymphocyte counts and decreases in serum lactate dehydrogenase (LDH) levels, two biomarkers that are associated with COVID-19 severity [18] and that are indicators of cytokine storm development [19]. Lymphocyte depression and fatigue, commonly observed in SARS-CoV-2 infection, occur secondary to overproduction of proinflammatory molecules (TNF-α, IL-6, IL-2, IL-10, and TNF-β) [20,21,22], while LDH has been described as a prognostic marker in several pulmonary conditions [23,24,25]. Despite no statistical significance in terms of mortality, days of hospitalization, or the need for intubation, the authors postulate that PTX administration could be of great value in the first-line care of COVID-19 due to its anti-inflammatory properties [18]. 

## 3. Pulmonary Fibrosis

Previous experiences from the SARS epidemic in 2002, with growing clinical evidence regarding the current pandemic, show that pulmonary fibrosis can be a long-term complication of SARS-CoV-2 infection [26]. Pulmonary fibrosis is a condition that potentially limits patients’ activities and lowers their quality of life due to a persistent alveolar restriction. Considering the mass spread of SARS-CoV-2 worldwide, residual pulmonary fibrosis may become a challenge for global healthcare and the economy. Fibrotic changes have been observed in patients that have recovered from SARS-CoV-2-related pneumonia in follow-up studies [27,28,29]. Post-COVID-19 fibrotic changes in the lung have also been reported in autopsy specimens [30,31]. Three main factors that make a patient more susceptible to the development of COVID-19-related pulmonary fibrosis are older age, severe pneumonia, and prolonged mechanical ventilation [32,33,34]. George et al. stated that some antifibrotic agents used in IPF treatment could be valuable in preventing post-COVID fibrosis or decreasing its severity [35]. 

The exact pathological mechanism of the development of fibrosis in COVID-19 is still not fully understood; thus, no specific treatment exists. The most probable pathomechanism involves overexpression of inflammatory cytokines, mainly as a result of alveolar damage, which later leads to the activation of fibroblasts and an over deposition of collagen in the pulmonary parenchyma [26,36,37]. One of the fibroblasts’ primary triggers is TGF-beta, a profibrotic cytokine that promotes the expression of other proinflammatory cytokines, such as TNF-α and IL-6 [36,38,39]. Reactive oxygen species (ROS) have also been shown to play an important role in the pathogenesis of pulmonary fibrosis, contributing to dysregulated proteostasis in the extracellular matrix and myofibroblast aging [40,41]. 

As PTX has been proven to possess anti-inflammatory effects, i.e., decreasing TNF-α, IL-6, and IL-1 plasma levels, as well the ability to decrease ROS production, and it has been widely evaluated in pre-clinical trials considering the treatment and prevention of various types of fibrosis. In animal models, PTX has shown efficacy in the prevention of renal interstitial fibrosis via inhibition of connective tissue growth factors and, thus, the prevention of extracellular matrix overproduction [42,43]. PTX–tocopherol combination treatment has been shown to reduce collagen type I and III depositions in the ECM in radiation-induced heart fibrosis using a rat model [44]. A small meta-analysis by Kaidar–Person et al. revealed limited, however promising, data on PTX-tocopherol use in radiation-induced fibrosis among patients undergoing radiotherapy for breast cancer [45]. In a rat model of radiation-induced lung injury, PTX inhibited fibrosis by reducing plasminogen activator inhibitor (PAI) and fibrinogen expression [46]. Some pre-clinical data also exists supporting the use of PTX in the treatment of peritoneal fibrosis due to its effects on attenuating TGF-beta and collagen production [47,48,49]. El-Lakkany et al. introduced the potential use of PTX as an adjuvant drug in schistosomal liver fibrosis; in the aforementioned paper, the authors propose downregulation of oxidative stress as the mechanisms behind the halt in fibrotic changes [50]. Additionally, in a study by Zabel et al., PTX was shown to inhibit TNF-α release from alveolar macrophages in patients with sarcoidosis, thus hindering the formation of sarcoid granulomas in the lung; some of the enrolled patients were also able to lower the dose of steroids in their treatment regimen [51]. These findings were later evaluated in a small, randomized controlled trial by Park et al., in which PTX was shown to reduce flares and exhibit a steroid-sparing effect in patients with sarcoidosis [52]. Nevertheless, PTX requires further investigations regarding this area due to the small sample sizes utilized.

Given all of the above data, we hypothesize that PTX may be a potent adjuvant drug for preventing and treating COVID-19-related pulmonary fibrosis. We call for large, population-based clinical trials repurposing this well-known drug with a previously established safety profile.

## 4. Coagulopathy

COVID-19 is a disease that, in its wide range of complications, does not spare hemostasis. Both thromboembolic and hemorrhagic events have been observed in SARS-CoV-2 infected patients, although the latter is much less common. Thrombi have occurred in multiple vascular beds, not limited to the alveolar vessels, and may lead to multi-organ failure. Iba et al., in a review article assessing 51 studies of coagulation in COVID-19, concluded that the endotheliopathy leading to lethal complications is mainly caused by direct viral injury to the vascular epithelium and a flourishing inflammatory response that results in vasculitis [53]. The most frequent biochemical findings considering the coagulation panel in COVID-19 are elevated D-dimer and fibrinogen serum levels, prolonged prothrombin times, and low platelet counts [54,55]. These alterations may appear somewhat similar to those observed in disseminated intravascular coagulation (DIC), a complication that develops in about 30–50% of severe sepsis patients, but the pathophysiology of COVID-associated coagulopathy is different from septic DIC [56]. DIC is characterized by a hypercoagulable state concomitant to excessive bleeding stemming from overconsumption of clotting factors [57]. Nevertheless, the D-dimer elevation in septic DIC is usually not as high as observed in COVID-19, and the platelet count drop in COVID-19 is not as profound as seen in DIC, which seems to be the reason why SARS-CoV-2 infection predisposes to thromboembolic complications [58]. The use of PTX in the treatment of DIC was evaluated by Ozden et al., who compared the effects of treatment with PTX and antithrombin III (ATIII) in 30 adult patients with Gram-negative sepsis who developed DIC; the authors concluded that both treatments were effective in terms of lowering D-dimer and fibrinogen levels, increasing platelet counts, and shortening prothrombin time (PT). Additionally, both PTX and ATIII lowered ISTH DIC scores with statistical significance [59]. Currently, PTX is mainly used in the treatment of intermittent claudication resulting from peripheral artery disease, in which it improves blood viscosity, stimulates fibrinolysis, makes erythrocytes more elastic and less prone to adhesion, and downregulates platelet aggregation [60,61,62,63]. Given this information, we may conclude that PTX is capable of improving blood flow and increasing tissue oxygenation. Thus in our opinion, PTX may be a powerful adjuvant therapeutic for COVID-19 patients, as it is able to diminish the effects of the hypercoagulable state via downregulation of the inflammatory response in vascular beds and can limit the tissue damage resulting from hypoxia.

## 5. Conclusions

Pentoxifylline (PTX) is a drug that exhibits broad-spectrum anti-inflammatory and immunomodulatory effects through mechanisms involving the adenosine A2A receptor (A2AR), in parallel with rheological effects. Previous studies have shown that PTX has anti-inflammatory effects and, therefore, may be beneficial in countering the cytokine storm caused by COVID-19. This drug has also been shown to reduce lung fibrosis in patients with COVID-19, as well as to prevent thromboembolic events. Therefore, PTX may exert potential benefits in treating the symptoms of SARS-CoV-2 as well as its complications. 

## Data Availability

Not applicable.
